# Altered Neuronal Intrinsic Properties and Reduced Synaptic Transmission of the Rat's Medial Geniculate Body in Salicylate-Induced Tinnitus

**DOI:** 10.1371/journal.pone.0046969

**Published:** 2012-10-10

**Authors:** Yan-Yan Su, Bin Luo, Yan Jin, Shu-Hui Wu, Edward Lobarinas, Richard J. Salvi, Lin Chen

**Affiliations:** 1 CAS Key Laboratory of Brain Function and Disease, School of Life Sciences, University of Science and Technology of China, Hefei, China; 2 Auditory Research Laboratory, University of Science and Technology of China, Hefei, China; 3 Department of Neuroscience, Carleton University, Ottawa, Ontario, Canada; 4 Center for Hearing and Deafness, State University of New York at Buffalo, Buffalo, New York, United States of America; University of Salamanca- Institute for Neuroscience of Castille and Leon and Medical School, Spain

## Abstract

Sodium salicylate (NaSal), an aspirin metabolite, can cause tinnitus in animals and human subjects. To explore neural mechanisms underlying salicylate-induced tinnitus, we examined effects of NaSal on neural activities of the medial geniculate body (MGB), an auditory thalamic nucleus that provides the primary and immediate inputs to the auditory cortex, by using the whole-cell patch-clamp recording technique in MGB slices. Rats treated with NaSal (350 mg/kg) showed tinnitus-like behavior as revealed by the gap prepulse inhibition of acoustic startle (GPIAS) paradigm. NaSal (1.4 mM) decreased the membrane input resistance, hyperpolarized the resting membrane potential, suppressed current-evoked firing, changed the action potential, and depressed rebound depolarization in MGB neurons. NaSal also reduced the excitatory and inhibitory postsynaptic response in the MGB evoked by stimulating the brachium of the inferior colliculus. Our results demonstrate that NaSal alters neuronal intrinsic properties and reduces the synaptic transmission of the MGB, which may cause abnormal thalamic outputs to the auditory cortex and contribute to NaSal-induced tinnitus.

## Introduction

Sodium salicylate (NaSal), an active metabolite of aspirin, is one of the most widely used analgesic, anti-inflammatory, and antipyretic drugs. High doses of NaSal have long been known to cause reversible tinnitus in patients [1], [2] and in animals [3], [4], [5], [6]. Because the clinical presentation of tinnitus is subjective, of unknown etiology, and variable, exploring the underlying mechanism of tinnitus in humans is difficult. To examine its biological bases, researchers frequently use NaSal to induce transient tinnitus in animal models [3], [5], [6], [7].

NaSal is believed to depress the neural output of the cochlea while cause hyperexcitability in some regions along the central auditory pathways [6], [8]. There is growing evidence that NaSal raises the excitability of the auditory cortex. For example, *in vivo* studies have shown that NaSal significantly increases both the sound-evoked [6], [9], [10] and spontaneous [11] neural activity in auditory cortex region. Fluorine-18 fluoro-2-deoxyglucose activity, a measure of metabolic/neuronal activation, increases in the rat auditory cortex after NaSal treatment [12]. Hyperexcitability in the auditory cortex is also reflected by increased numbers of c-Fos immunoreactive neurons after NaSal treatment [13]. Our previous work on slices of the auditory cortex demonstrated that NaSal reduced the inhibitory postsynaptic current (IPSC) [14] and selectively suppressed the current-evoked firing of GABAergic interneurons without affecting glutamatergic pyramidal neurons [15]. Both of these effects increase the excitability of the auditory cortex by altering the balance between excitation and inhibition. Collectively, these results suggest that increased neural excitability in the auditory cortex may be a key mechanism underlying NaSal-induced tinnitus [16], [17]. NaSal also increases neuronal excitability in the rat hippocampus. It does so by reducing GABAergic inhibition without affecting intrinsic membrane excitability in CA1 pyramidal neurons [18]. The NaSal-induced increase in hippocampal excitability may contribute to non-auditory aspects of tinnitus because the limbic system has been implicated in tinnitus perception [17], [19], [20], [21]. The ability of NaSal to elevate the excitability in the cerebral cortex (e.g., the auditory cortex and the hippocampus) raises a question of whether NaSal exerts the same effect in subcortical brain structures, which may be part of a neural network involved in the perception of tinnitus.

The medial geniculate body (MGB) is the part of the classical auditory pathway, which provides the primary and immediate inputs to the auditory cortex. The MGB has extensive afferent and efferent connections with the auditory cortex and plays a key role in auditory perception [22], [23]. In this way, it makes an appropriate candidate for the study of the neural mechanisms underlying tinnitus. To date, only a few studies have examined the effect of NaSal on neural activity in the MGB [12], [24], and the data are limited at the cellular level. To address this issue, we investigated how NaSal modulates the intrinsic membrane properties and synaptic responses of MGB neurons using whole-cell patch-clamp recordings from brain slices. In addition, we used the gap prepulse inhibition of acoustic startle (GPIAS) paradigm to confirm that salicylate induces tinnitus-like behavior in rats [6], [25].

## Results

### NaSal induced tinnitus-like behavior in rats

**Figure 1:**
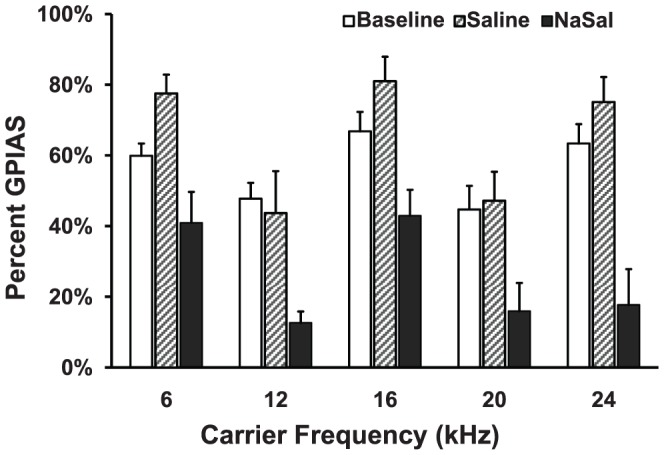
Gap prepulse inhibition of acoustic startle (GPIAS) as a function of carrier frequency for saline and sodium salicylate (NaSal) treatments relative to baseline. Saline treatment had no effect on GPIAS performance (*n* = 6, *P*>0.05). In contrast, treatment of NaSal at a dose of 350 mg/kg caused a significant reduction in GPIAS performance (*n* = 6, *P*<0.001). Data are the mean ± SEM. (two-way repeated measures analysis of variance, two way RM-ANOVA).

Tinnitus was evaluated in 6 rats following treatment with saline and a 350 mg/kg dose of NaSal. NaSal caused a statistically significant decrease in GPIAS values relative to baseline across multiple frequencies (Fig. 1). This indicated that these rats were experiencing tinnitus. A two-way repeated measures analysis of variance (RM-ANOVA) with main effect treatment (factor 1) and frequency (factor 2) indicated a significant difference between NaSal treatment and baseline (*P*<0.001) and a significant difference between NaSal treatment and saline (*P*<0.001). However, there was no difference between baseline and saline treatments (*P*>0.05) indicating that saline treatment had no effect on GPIAS at these frequencies. The post hoc Tukey's test revealed a statistically significant frequency effect between NaSal treatment and baseline GPIAS values at 12, 16, 20 and 24 kHz, demonstrating that NaSal induces tinnitus over a broad range of high frequencies.

### NaSal decreased membrane input resistance and hyperpolarized MGB neurons

**Figure 2:**
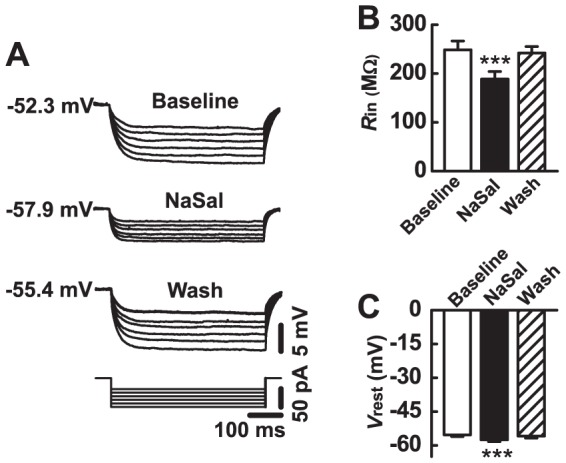
Effects of NaSal on membrane input resistance (*R*_in_) and resting membrane potential (*V*_rest_) of neurons in the medial geniculate body (MGB). (A) A series of hyperpolarizing current ranging from −30 to −80 pA (−10 pA/step) was injected into a MGB neuron for 500 ms before (Baseline), during (NaSal), and after (Wash) application of 1.4 mM NaSal. The *V*_rest_ is indicated beside the traces. (B) Mean *R*_in_ (*n* = 23) and (C) *V*_rest_ (*n* = 28) before (Baseline), during (NaSal), and after (Wash) application of 1.4 mM NaSal in the MGB neuron. Data are the mean ± SEM. ***P<0.001 relative to baseline (one-way ANOVA with Bonferroni correction, 3 pairwise comparisons).

To determine how NaSal affects the membrane excitability of MGB neurons, we applied NaSal in the bath solution during current-clamp recording. Fig. 2A shows sample traces of membrane potentials recorded from a MGB neuron following step current injections from which the membrane input resistance was derived. Group data show that NaSal significantly decreased the membrane input resistance from 248.8±18.1 MΩ to 188.8±15.4 MΩ (*n* = 23, *P*<0.001) (Fig. 2B). This decrease in the membrane input resistance was reversible following washout (*P*>0.05). NaSal also significantly hyperpolarized the resting membrane potential from −55.3±0.8 mV to −57.5±0.7 mV (*n* = 28, *P*<0.001) (Fig. 2A and C). This hyperpolarizing effect was also reversible following washout (*P*>0.05).

### NaSal suppressed the current-evoked firing and changed the action potential in MGB neurons

**Figure 3:**
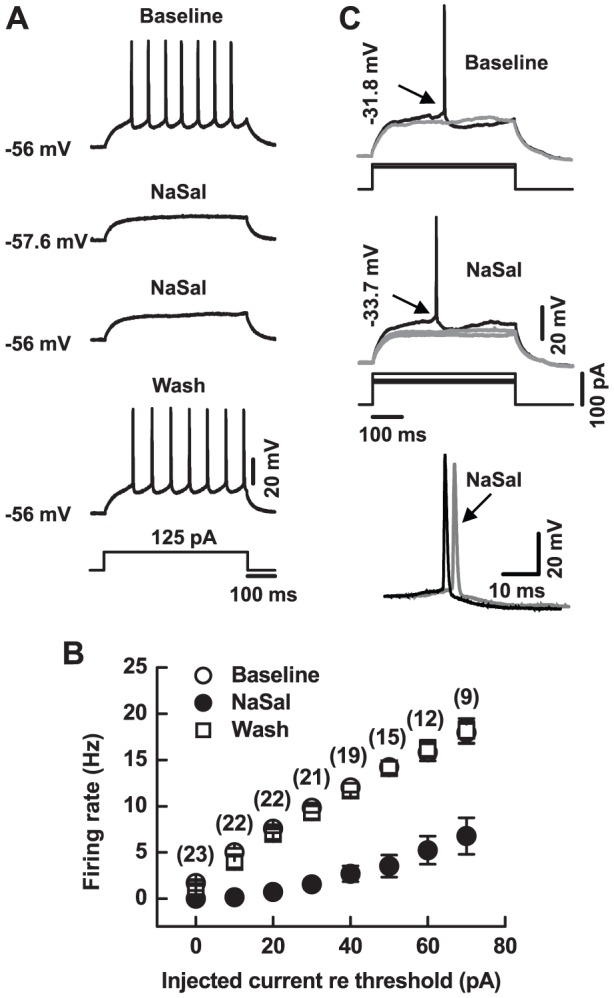
Effects of NaSal on current evoked firing and the action potential properties of MGB neurons. (A) A 125 pA current was injected into a MGB neuron for 500 ms before (Baseline), during (NaSal), and after (Wash) application of 1.4 mM NaSal. Note that the neuron's resting membrane potential was adjusted to the baseline levels during NaSal exposure (third trace from the top). (B) Mean evoked firing rate at all superthreshold levels before (Baseline), during (NaSal), and after (Wash) application of 1.4 mM NaSal in MGB neurons. Treatment with NaSal caused a significant reduction in evoked firing rate (*P*<0.001). Sample sizes are indicated in parentheses (two-way ANOVA). (C) Upper traces: responses of a MGB neuron to 70 pA (gray) and 80 pA (black) current injections for 500 ms before NaSal. Middle traces: responses of the same neuron to 80 and 90 pA (gray) and 100 pA (black) current injections for 500 ms during NaSal. Lower traces: action potential waveforms shown in upper (black) and middle (gray) traces are overlapped. The thresholds for generation of action potentials are indicated by arrows.

Electrical currents at various levels up to 70 pA above the threshold level were injected into MGB neurons to evoke action potential firing. NaSal reversibly decreased the firing rates in all 23 neurons tested (Fig. 3). Among these neurons, firing of 13 neurons evoked by current injections at these various levels was completely blocked by NaSal (Fig. 3A, upper two traces). Two-way ANOVA revealed that NaSal significantly decreased the evoked firing rate of the neurons at current levels up to 70 pA above the threshold current (*n* = 9–23 for different current levels, *P*<0.001) (Fig. 3C). To rule out the possibility that the suppression of firing was due to the membrane hyperpolarization caused by NaSal, we adjusted the resting membrane potential equal to the level before drug application in 4 neurons during their exposure to NaSal to determine whether the depression persisted. The firing remained suppressed after the adjustment, as illustrated in Fig. 3A (third trace from the top). Neither the change in resting membrane potential (*n* = 19, *P* = 0.089, *r^2^* = 0.16) nor the change in membrane input resistance (*n* = 19, *P* = 0.067, *r^2^* = 0.183) was correlated with the change in firing rate (at 40 pA re threshold level), indicating that other mechanisms rather than resting membrane potential or membrane input resistance are involved in the effects of NaSal on the evoked firing.

The action potential properties of the 10 neurons that generated spikes during NaSal treatment were also affected. Fig. 3C shows sample action potentials in the presence and absence of NaSal. For this neuron, the action potential threshold was increased by ≈2 mV and the threshold current was increased by 20 pA (Before NaSal, 80 pA induced one action potential, and after NaSal, 100 pA induced one action potential) (Fig. 3C, top and middle traces). The amplitude, rise and decay time, and half-width of action potential were all affected by NaSal (Fig. 3C, bottom trace). Table 1 shows that, during NaSal treatment, the action potential amplitude (*n* = 10, *P*<0.01), rise slope (*n* = 10, *P*<0.05), and decay slope (*n* = 10, *P*<0.01) were all reduced significantly, and the action potential threshold (*n* = 10, *P*<0.05), half-width (*n* = 10, *P*<0.05), and the threshold current (*n* = 10, *P*<0.001) increased significantly. However, the afterhyperpolarization (AHP) remained unchanged (*n* = 10, *P*>0.05).

**Table 1:** Effects of 1.4 mM sodium salicylate (NaSal) on action potential properties (*n* = 10).

Measurements	Before NaSal perfusion			After NaSal perfusion		
Threshold (mV)^1^	−31.3	±	1.4	−29.3	±	1.6*
Amplitude (mV)^2^	68.0	±	2.7	63.4	±	2.8**
Rise slope (mV/ms)	83.4	±	11.9	72.4	±	11.9*
Decay slope (mV/ms)	−32.6	±	3.1	−29.0	±	2.7**
Half-width (ms)	1.5	±	0.1	1.6	±	0.1*
Threshold current (pA)^3^	44.5	±	6.7	77.5	±	11.8***

All data are expressed as means ± SEM. *, ** and *** indicate *P*<0.05, *P*<0.01 and *P*<0.001, respectively (paired Student's *t*-test).

1 The action potential threshold was defined as the membrane potential at which the potential of the membrane began to rise rapidly and the neuron generated only one or two spikes.

2 The action potential amplitude was calculated by subtracting the threshold from the peak value of the action potential.

3 The threshold current was defined as the minimum current that is required to elicit one or two spikes.

### NaSal depressed the rebound depolarization in MGB neurons

**Figure 4:**
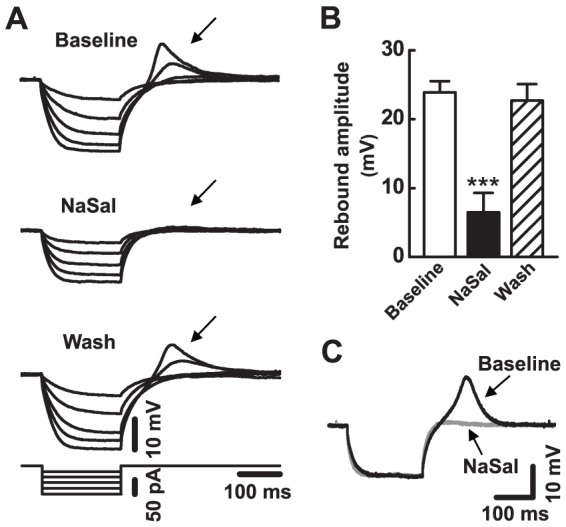
Effects of NaSal on rebound depolarization in MGB neurons. (A) A series of hyperpolarizing current ranging from −20 pA to −100 pA (20 pA/step) was injected into a MGB neuron before (Baseline), during (NaSal), and after (Wash) application of 1.4 mM NaSal. The rebound depolarization following membrane hyperpolarization is indicated with an arrow. (B) The mean amplitude of rebound depolarization before (Baseline), during (NaSal), and after (Wash) application of 1.4 mM NaSal in the MGB neurons (*n* = 10). Data are the mean ± SEM. ***P<0.001 relative to baseline (one-way ANOVA with Bonferroni correction, 3 pairwise comparisons). (C) Response of another neuron to −80 pA current injection in ACSF (black trace) and to −100 pA current injection in 1.4 mM NaSal (gray trace).

Almost all of the MGB neurons tested had a rebound depolarization (Fig. 4A), which consisted of depolarization immediately following membrane hyperpolarization [26], [27], [28]. NaSal reversibly reduced the rebound in all 10 neurons tested. Fig. 4A shows sample traces of rebound potentials recorded from a MGB neuron in the presence and absence of NaSal. The amplitude of rebound depolarization was gradually and drastically decreased from 23.9±1.6 mV to 6.5±2.8 mV by NaSal (*n* = 10, *P*<0.001) (Fig. 4B).

Note that the membrane input resistance was also decreased by NaSal (Fig. 4A middle panel). Because the amplitude of the rebound depolarization would decrease if the magnitude of the preceding hyperpolarization were smaller [29], the reduction in the rebound might be due to smaller pre-hyperpolarization induced by NaSal. To rule out this possibility, we injected larger negative currents into 3 neurons during NaSal treatment in order to match the hyperpolarization to the level observed in the absence of NaSal. Under these conditions, NaSal still suppressed the rebound (Fig. 4C). No correlation was observed between the change in rebound amplitude and the change in membrane input resistance (*n* = 10, *P* = 0.384, *r^2^* = 0.096), indicating that NaSal had a direct action on the rebound.

### NaSal depressed evoked postsynaptic responses in MGB neurons

**Figure 5:**
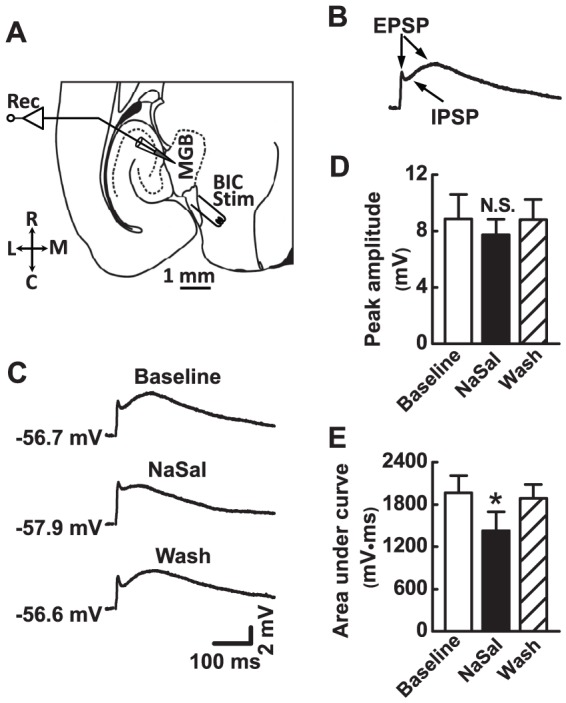
Effects of NaSal on postsynaptic responses of MGB neurons evoked by stimulating the brachium of the inferior colliculus (BIC). (A) A schematic drawing of a brain slice with horizontal section of the MGB. Locations of recording (Rec) and electrical stimulation (Stim) are illustrated. R, rostral; C, caudal; L, lateral; M, middle. (B) Sample trace of the typical postsynaptic potential (PSP) recorded. The PSP had two components: excitatory postsynaptic potential (EPSP) and inhibitory postsynaptic potential (IPSP). The trace was averaged from 6 consecutive sweeps. (C) Representative traces of PSP before (Baseline), during (NaSal), and after (Wash) application of 1.4 mM NaSal in a MGB neuron. Each trace was averaged from 6 consecutive sweeps. The resting membrane potential is indicated beside the traces. (D) Mean peak amplitude of the PSP (*n* = 9) and (E) area under PSP curve (*n* = 9) before (Baseline), during (NaSal), and after (Wash) application of 1.4 mM NaSal in the MGB neuron. Data are the mean ± SEM. *P<0.05, ^N.S.^ *P*>0.05 relative to baseline (one-way ANOVA with Bonferroni correction, 3 pairwise comparisons).

To evoke postsynaptic responses in MGB neurons, we electrically stimulated the brachium of the inferior colliculus (BIC), which contains colliculogeniculate axons (Fig. 5A). The evoked postsynaptic activity was recorded in current-clamp mode without application of receptor antagonists. Among 11 neurons we recorded, 6 neurons had a mixture of excitatory postsynaptic responses and inhibitory postsynaptic responses (Fig. 5B) [26] whereas the others either had a unitary excitatory response (3 out of 11 neurons) or had a unitary inhibitory response (2 out of 11 neurons). Fig. 5C shows sample traces of mixed postsynaptic responses recorded from a MGB neuron in the absence and presence of NaSal. There is no significant difference in the peak amplitude of postsynaptic responses with excitatory components (i.e., the mixed responses together with the unitary excitatory responses) before and after application of NaSal (8.9±1.8 vs. 7.7±1.1 mV, *n* = 9, *P*>0.05) (Fig. 5D). However, NaSal significantly and reversibly decreased the response area above the resting membrane potential from 1965.6±264.6 to 1428.1±194.5 mV•ms (*n* = 9, *P*<0.05) (Fig. 5E).

### NaSal depressed the postsynaptic responses mediated by GABA_A_ or NMDA receptors, but not AMPA receptors in MGB neurons

The fast postsynaptic responses recorded from MGB neurons are mediated mainly by GABA_A_, NMDA, and AMPA receptors [26], [28]. We were interested in which receptors NaSal acts on for the reduction of the postsynaptic responses. To address this issue, we took voltage-clamp recordings of MGB neurons with specific antagonists added to ACSF or the pipette solution. We also clamped the membrane potentials to specific levels in order to distinguish the postsynaptic responses mediated by different receptors.

**Figure 6:**
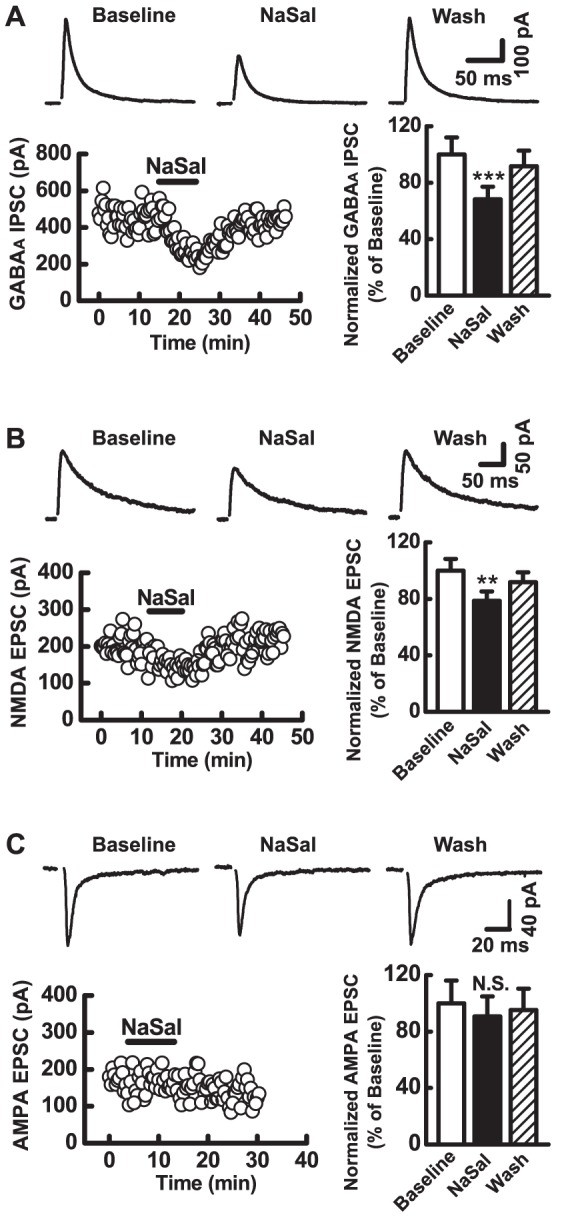
Effects of NaSal on the GABA_A_, the NMDA, and the AMPA receptor-mediated postsynaptic current of MGB neurons evoked by stimulating the BIC. Upper panels in (A), (B) and (C): representative traces of GABA_A_ receptor-mediated inhibitory postsynaptic current (IPSC), NMDA, and AMPA receptor-mediated excitatory postsynaptic current (EPSC) before (Baseline), during (NaSal), and after (Wash) application of 1.4 mM NaSal in a MGB neuron, respectively. Each trace was averaged from 6 consecutive sweeps. Lower left panels in (A), (B) and (C): time course of the amplitude of IPSCs and the EPSCs recorded in the same neuron as in each upper panel with application of 1.4 mM NaSal (horizontal bar). Lower right panels in (A), (B) and (C): mean amplitude of GABA_A_ receptor-mediated IPSCs (*n* = 22), NMDA receptor-mediated EPSCs (*n* = 18) and AMPA receptor-mediated EPSC (*n* = 15) before (Baseline), during (NaSal), and after (Wash) application of 1.4 mM NaSal in the MGB neurons. Data are the mean ± SEM. ***P<0.001, **P<0.01, ^N.S.^ *P*>0.05 relative to baseline (one-way ANOVA with Bonferroni correction, 3 pairwise comparisons).

To determine the IPSC mediated by GABA_A_ receptors, we blocked ionotropic glutamatergic receptors by adding 4 mM of kynurenic acid to the bath solution and maintained the membrane potential at 0 mV. NaSal gradually and reversibly decreased the amplitude of GABA_A_ receptor-mediated IPSCs by 31.8±8.9% of the control (*n* = 22, *P*<0.001) (Fig. 6A). To determine the excitatory postsynaptic current (EPSC) mediated by NMDA receptors, we blocked GABA_A_ receptors and AMPA receptors by adding picrotoxin (100 µmol/L) and CNQX (10 µmol/L) to the bath solution and held the membrane potential at +40 mV to relieve the voltage-dependent Mg^2+^ blockade on the NMDA receptor channels. NaSal gradually and reversibly decreased the amplitude of NMDA receptor-mediated EPSCs by 21.3±6.7% of the control (*n* = 18, *P*<0.001) (Fig. 6B). To determine the EPSC mediated by AMPA receptors, we blocked GABA_A_ receptors by adding picrotoxin (100 µmol/L) to the bath solution and minimized NMDA receptor-mediated components by maintaining the membrane potential at −80 mV. NaSal did not significantly change the amplitude of AMPA receptor-mediated EPSCs (Fig. 6C) (*n* = 15, *P*>0.05).

## Discussion

Using a GPIAS paradigm, we successfully demonstrated that a high dose of NaSal (350 mg/kg) can reliably induce tinnitus-like behavior in Wistar rats (Fig. 1). This is consistent with previous reports using other strains [3], [6], [25], [30], [31]. To explore the underlying neural mechanisms of this tinnitus-like behavior in rats, we examined effects of NaSal on the neuronal and synaptic responses in rat MGB *in vitro*. The neurons we recorded were likely from two main subdivisions of the MGB, the dorsal and ventral MGB because of their characteristic electrophysiological properties (i.e., the monophasic AHPs and rebound bursts, data not shown) [32]. We have found that (1) NaSal changed the intrinsic properties of MGB neurons, such as the membrane input resistance (Fig. 2), the resting membrane potential (Fig. 2), the current-evoked firing (Fig. 3), the shape of the action potential (Fig. 3, Table 1) and the rebound depolarization (Fig. 4); (2) NaSal reduced the postsynaptic response evoked by stimulating the BIC. The altered intrinsic neuronal properties and reduced synaptic transmission by NaSal suggest a possible role of the MGB in NaSal-induced tinnitus.

### NaSal may alter the intrinsic properties of MGB neurons by targeting membrane ion channels

It is likely that NaSal alters the intrinsic properties of MGB neurons through its actions on the membrane ion channels expressed in the MGB neurons. In the present study, NaSal significantly reduces the membrane input resistance of MGB neurons and hyperpolarizes their resting membrane potentials (Fig. 2). This suggests that NaSal influences the ion channels that maintain the resting membrane potential, such as the KCNK channels [33], [34]. Although the detailed mechanisms are not fully understood, one possibility is that NaSal causes more K^+^ ions to leak out of the cell, thereby reducing the membrane resistance and hyperpolarizing the membrane potential. NaSal was demonstrated in inferior colliculus neurons to modulate the function of voltage-gated Na^+^ channels [35], which mediate the rapidly rising phase and initial component of the falling phase of an action potential [36], [37]. By blocking the voltage-gated Na^+^ channels in the MGB neurons, NaSal could reduce the evoked firing rate, elevate action potential threshold, decrease action potential amplitude, reduce the action potential's rise and decay slopes, and prolong the action potential half-width as we observed in the present study (Fig. 3, Table 1). In the MGB, rebound depolarization is mediated by the low-threshold Ca^2+^ channels [26], [27], [28]. The present study shows that NaSal directly depresses the rebound depolarization (Fig. 4), suggesting that NaSal may also have a depressive effect on these low-threshold Ca^2+^ channels. The notion that NaSal changes the intrinsic properties of MGB neurons through its actions on the membrane ion channels needs to be confirmed using voltage-clamp recordings.

### How does NaSal reduce the afferent synaptic transmission in the MGB?

The present study shows that NaSal depressed postsynpatic responses in MGB neurons to the stimulation of the BIC. Because the BIC contains colliculogeniculate axons, the evoked postsynaptic responses recorded in the MGB neurons simulated the afferent synaptic inputs from the inferior colliculus. These fast postsynaptic responses are mediated mainly by GABA_A_, NMDA, and AMPA receptors [26], [28]. In the present study, the IPSC mediated by GABA_A_ receptors was depressed significantly by NaSal. In addition, the EPSC mediated by NMDA receptors, but not by AMPA receptors, was depressed by NaSal (Fig. 6). These results indicate that NaSal reduces both inhibitory and excitatory postsynaptic responses mainly through its actions on GABA_A_ and NMDA receptors.

Although NaSal suppressed both inhibitory and excitatory postsynaptic responses in the MGB, the inhibitory component of postsynaptic currents was suppressed to a more pronounced degree (Fig. 6A) than the excitatory component (Fig. 6B). However, the summed postsynaptic response consisting of inhibitory and excitatory components was not potentiated by NaSal as a net effect (Fig. 5E). This is probably because there was a difference in the number between excitatory and inhibitory fibers of the BIC that were recruited by electrical stimulation. If more excitatory fibers are activated, NaSal would suppress EPSCs more than IPSCs, resulting in less excitation than inhibition. In this way, no potentiation of the overall synaptic responses would take place. In addition, the decreased membrane input resistance, hyperpolarized resting membrane potential, and cross-talk between NMDA and GABA_A_ receptors [38] may also contribute to the suppressive effect of NaSal on the synaptic response.

### NaSal likely lowers the excitability of the MGB

The altered neuronal intrinsic properties (Figs. 2, 3, 4) as well as reduced synaptic transmission (Figs. 5 and 6) by NaSal suggests reduced neuronal excitability of the MGB in rats following treatment of NaSal. The specific evidence supporting this notion includes: (1) the decrease in the membrane input resistance together with the hyperpolarized resting membrane potential caused by NaSal would lower neuronal excitability; (2) the suppressed probability of generating action potentials by NaSal is a reflection of the reduced membrane excitability of MGB neurons after NaSal perfusion; (3) rebound depolarization is believed to enhance membrane excitability and promote the production of action potentials [29], [39]. If this is correct, then the depression of the rebound by NaSal may acts as another mechanism by which NaSal suppresses neuronal excitability; (4) the reduction in postsynaptic responses evoked by BIC stimulation as a net effect after NaSal perfusion indicates that NaSal reduces the efficacy of synaptic transmission from the inferior colliculus to MGB neurons (Fig. 5). In rat MGB, GABAergic neurons are in low abundance (<1%) [40], [41] although there are morphologically different cell types [42], [43]. Thus, most neurons we recorded in the MGB were likely glutamatergic and the reduced neuronal excitability of these presumably glutamatergic neurons by NaSal suggests lowered excitability of the MGB.

NaSal can penetrate the blood-brain barrier and reach concentrations up to 310 mg/L (1.94 mM) in the cerebrospinal fluid of animals treated with high doses of NaSal [44]. Consequently, when it is systemically applied, NaSal not only affects the cochlea but can directly affect the excitability of broad brain regions within and outside of the central auditory system including the MGB to generate tinnitus. We have previously demonstrated that NaSal (1.4 mM) selectively depressed the firing rate of GABAergic fast-spiking interneurons without affecting the firing rate of pyramidal neurons in the auditory cortex [15]. In other areas of the forebrain, such as the hippocampus, NaSal also exerted an excitatory effect [18]. In the inferior colliculus, NaSal induces an increase of the spontaneous activity [45], [46]. However, in the dorsal cochlear nucleus, NaSal suppresses spontaneous and evoked firing in fusiform cells but has little effect on the firing of glycinergic cartwheel cells [47]. In the MGB, the neural excitability is likely lowered by NaSal as illustrated in the present study. Taken together, these results indicate that the effects of NaSal on neural activity are remarkably dependent on brain region. Why NaSal has such widely varied effects on different auditory structures remains largely a mystery at this point. Discovering the reasons for the brain-region-dependent actions of NaSal may have important implications for our understanding of the neural mechanisms underlying tinnitus.

### Role of the MGB in NaSal-induced tinnitus

The MGB provides the primary and immediate inputs to the auditory cortex. Paradoxically, we found that NaSal likely lowers the excitability of the MGB but increases the excitability of neurons in the auditory cortex [9], [15]. If the context of the neural networks mediates the perception of tinnitus, then there is a question as to how NaSal-induced decreases in MGB excitability contribute to the generation of tinnitus-like behavior in rats. We speculate that the decreased excitability of the MGB will lead to reduced thalamic inputs to the auditory cortex. Because these inputs have been shown to activate inhibitory interneurons more strongly than excitatory neurons in the neocortex [48], the reduced inputs by NaSal would presumably change the inhibition-excitation balance towards excitatory side, resulting in an increase in excitability in the neural networks of the auditory cortex. In this sense, the MGB plays a role in generation of NaSal-induced tinnitus.

### Difference in the age between animals used in behavior and *in vitro* experiments

One of limitations of the present study is that young animals (15.8±0.2 postnatal days) were used for the patch-clamp experiment whereas adult animals (3–5 months old) were used in the behavioral experiment. Although there is evidence to show that the intrinsic properties (e.g. the resting membrane potential and the membrane input resistance) of MGB neurons do not change very much from 16 to 21 postnatal days in rats [49], [50], caution should still be taken when the results obtained in the patch-clamp experiment are used to interpret those in the behaviral experiment.

## Materials and Methods

### Subjects

Adult male Wistar rats (3–5 months old, 325–450 g) were used to assess NaSal-induced tinnitus using GPIAS. Young Wistar rats of both sexes (13–21 postnatal days; average age slices were recorded from is 15.8±0.2 postnatal days) were used for patch-clamp experiments. The behavioral experimental procedures used in the present study were approved by the University at Buffalo Institutional Animal Care and Use Committee. The experimental procedures for the patch-clamp experiments were in accordance with the protocols approved by the Institutional Animal Care and Use Committee of University of Science and Technology of China. All efforts were made to minimize the number of animals used and their suffering.

### Behavioral measures of NaSal-induced tinnitus

Tinnitus was assessed using the GPIAS paradigm as described in detail in previous reports [6], [25]. This procedure utilized the acoustic startle reflex test in animals treated with NaSal. Baseline measures of GPIAS were collected first, followed by treatment with saline (2.2–3.2 ml i.p.). Following a 5 day washout period, rats were treated with NaSal (350 mg/kg, 2.2–3.2 ml saline vehicle, 50 mg/ml concentration, i.p.). GPIAS testing began 1 hour after administration of either saline or NaSal. Each rat was placed in an acoustically transparent wire-mesh cage mounted on Plexiglas base which rested on a sensitive piezoelectric transducer that generated a voltage proportional to the magnitude of the startle response evoked by sound stimuli generated digitally by digital signal processor (Tucker Davis, TDT RX6, U.S.). The output of the startle platform was amplified, sampled, and stored on a computer for offline analysis.

GPIAS sessions were composed of 100 gap trials and 100 no-gap trials. Twenty measurements were taken at each noise-band center frequency (narrow band noise centered at 6, 12, 16, 20, or 24 kHz). Gap and no-gap trials were presented in random pairs. Trials were separated by a variable intertrial interval of 7–15 s. A 2 minute acclimation period occurred at the beginning of each session during which no stimuli were presented. Gap trials started with a background of narrow band noise centered at 6, 12, 16, 20, or 24 kHz (≈60 dB SPL, BW = 100–5000 Hz). During each gap trial, a brief, silent 75 ms gap was inserted 100 ms prior to the startling stimulus (5–10 kHz bandpass noise, 105 dB SPL). No-gap trials were identical to gap trials except that the silent gaps were omitted from the trials.

### Preparation of brain slices

On the day of patch-clamp experiment, the brain slices of MGB were prepared as described previously [51]. Briefly, the animal was decapitated and the brain was carefully taken out. Using a vibrating microtome (VT-1000S; Leica, Germany), three or four 290–390 µm thick horizontal slices including MGB were obtained from the brain. After at least 1 hour of incubation in oxygenated (95% O_2_ and 5% CO_2_) artificial cerebrospinal fluid (ACSF) at 26°C, one slice was transferred to a submerged recording chamber that was continuously perfused (3 ml/min) with oxygenated ACSF. The temperature of the bath solution was monitored and maintained at 25–26°C.

### Solutions and drugs used for patch-clamp recording

The composition of the standard ACSF was (in mM): NaCl 124; KCl 5; MgSO_4_ 1.3; KH_2_PO_4_ 1.2; glucose 10; NaHCO_3_ 24; CaCl_2_ 2.4 (pH: 7.4, osmolarity: 290–300 mOsm/L). The composition of the pipette solution was (in mM): K-gluconate 130; MgCl_2_ 2; KCl 5; EGTA 0.6; HEPES 10; Na-GTP 0.3; Mg-ATP 2 (pH: 7.2, osmolarity: 280 mOsm/L) for current-clamp recording and (in mM): Cs-methanesulfonate 130; CaCl_2_ 0.15; MgCl_2_ 2.0; EGTA 2.0; HEPES 10; Na_2_-ATP 2.0; Na_3_-GTP 0.25; QX-314 10 (pH: 7.2, osmolarity: 282 mOsm/L) for voltage-clamp recording. NaSal was dissolved in ACSF just before use and the concentration of NaSal we used was 1.4 mM, the typical concentration found in the cerebrospinal fluid of animal models with NaSal-induced tinnitus [52], [53]. After stable baseline responses were acquired, NaSal was normally administrated for 7–10 min when the reactions to the drug were most prominent. All drugs used in this study were purchased from Sigma Aldrich, Co. (St. Louis, MO, U.S.).

### Whole-cell patch-clamp recording and electrical stimulation

Patch pipettes were pulled from glass capillaries with an outer diameter of 1.5 mm on a two-stage puller (PC-10; Narishige, Tokyo, Japan). The resistance of the recording electrode filled with pipette solution was 3–5 MΩ. A patch-clamp amplifier (EPC9; HEKA Electronics, Germany) and a built-in PCI-16 interface board were used for whole-cell patch-clamp recordings. The MGB neurons were visualized under a 40× water immersion objective on an upright microscope (E-600-FN; Nikon, Japan) equipped with an infrared camera. Data were sampled using a computer installed with Pulse software (Version 8.80; HEKA Electronics, Germany). Only those neurons with series resistance <30 MΩ and input resistance >100 MΩ were included in this study. If the series resistance changed by more than 20% of the initial value during the recording, the data was discarded.

To evoke postsynaptic responses, we placed a bipolar stimulating electrode consisted of two tungsten wires separated by ∼500 µm on the BIC just caudal to the MGB (Fig. 5A). A single 100–200 µs rectangular electrical pulse was generated by a stimulator (SEN-7203; Hikon Kohden, Japan) and delivered at 0.05 Hz through an isolation unit (SS-202J; Hikon Kohden, Japan). The electrical stimulation to the BIC, which contains the colliculogeniculate axons, simulated the neural inputs from the inferior colliculus to the MGB. The strength of stimulation was set to a level at which the EPSC or IPSC amplitude was about 50–70% of the maximum amplitude evoked.

### Data analysis

GPIAS was calculated by computing the average ratio of trials with a gap versus trials with no-gaps for each frequency using the formula: GPIAS = (1−(AvgT_gap_/AvgT_nogap_))×100%; where AvgT_gap_ is the average amplitude during gap trials, and AvgT_nogap_ is the average amplitude of trials with no gap. The data were analyzed using a two-way RM-ANOVA to determine the main effects of treatment and the interaction between treatment and frequency, and post-hoc Tukey's tests were performed to make multiple comparison on different frequencies (SigmaStat 3.5 software).

For patch-clamp experiments, all the measurements were made from the recordings at least 5–10 min after establishing a whole-cell configuration and showing a stable resting membrane potential. The methods for data analysis of intrinsic membrane properties and synaptic responses were similar to those described previously [54]. Changes in membrane potential elicited by intracellular current injection (−30 to −80 pA) were measured between the baseline membrane potential and the peak hyperpolarization. The current-voltage (*I–V*) curve was plotted and then the slope was calculated from the linear range of the curve. The value of the *I–V* curve slope was defined as the input resistance of the cell membrane. The action potential threshold was defined as the membrane potential at which the potential of the membrane began to rise rapidly and the neuron generated only one or two spikes. The threshold current for firing was defined as the minimum amplitude of current injection required to elicit at least one or two spikes. The amplitude of an action potential was defined as the difference between the action potential threshold and the peak voltage of the action potential. The amplitude of the rebound depolarization following membrane hyperpolarization was defined as the difference between the resting membrane potential and the peak of the rebound.

Off-line data analysis for patch-clamp experiments was carried out using Pulse software version 8.80 (HEKA Electronic, Germany), Clampfit software version 9.2 (Axon Instruments Inc, U.S.), and MiniAnalysis software version 6.03 (Synaptosoft Inc, U.S.). For the purpose of statistical analysis, the data from the same neuron were averaged samples within a 2 min time window and collected before (baseline recording), during (NaSal exposure), and after (wash) NaSal application. The processed data were imported into Origin software version 7.5 (OriginLab Corporation, U.S.) for generating graphs. The statistical significance of differences between two groups was determined using paired Student's *t*-tests. For multiple group comparisons, statistical significance was determined using one-way analysis of variance (ANOVA) with Bonferroni correction or two-way ANOVA. All data are expressed as means ± SEM, where *n* represents the number of neurons. Two-sided *P*≤0.05 was regarded as statistically significant.
